# Repeatability of Clinical, Biomechanical, and Motor Control Profiles in People with and without Standing-Induced Low Back Pain

**DOI:** 10.1155/2010/289278

**Published:** 2010-07-18

**Authors:** Erika Nelson-Wong, Jack P. Callaghan

**Affiliations:** ^1^School of Physical Therapy, Regis University, 3333 Regis Blvd. G-4, Denver, CO 80221, USA; ^2^Department of Kinesiology, Faculty of Applied Health Sciences, University of Waterloo, Waterloo, Ontario, Canada N2L 3G1

## Abstract

A major research focus is optimization of interventions for low back pain (LBP). Predisposing factors for LBP development have been previously identified. To differentiate changes in these factors with intervention, factor stability over time must be determined. Twenty-three volunteers without LBP participated in a LBP-inducing standing protocol on two separate days. Outcome measures included visual analog scale (VAS) for LBP and trunk/hip muscle coactivation patterns. Intraclass correlation coefficients (ICCs) were used to examine repeatability. Between-day repeatability of outcome measures was excellent (ICCs >0.80). Individuals were consistent in subjective LBP, with 83% reporting similar day-to-day VAS levels. Muscle co-activation patterns and LBP reports are stable measures over time for this LBP-inducing protocol. Changes in these measures following intervention can be considered to be treatment effects and are not due to natural variability. This provides support for use of this protocol in studying interventions for standing-induced LBP.

## 1. Introduction/Background

Low back pain (LBP) is a pervasive problem that exacts many socioeconomic and personal costs each year [1, 2]. Many current musculoskeletal research efforts in biomechanics and kinesiology are focused on the identification of predictive factors for LBP development, and evaluating specific motor pattern responses to different interventions. As factors that are thought to be associated with or predictive of LBP development are identified and characterized, interventions can be targeted towards changing or modifying these factors, with the ultimate goal of improving LBP intervention effectiveness. In order to confidently differentiate changes in motor patterns in response to an intervention from natural variability, however, the normal day-to-day stability of the motor patterns of interest must first be established.

Multiple studies have demonstrated that exposure to prolonged standing substantially increases the risk of experiencing LBP [3–5]. A prolonged standing protocol has been useful in identification of predisposing factors for LBP and evaluating the response of these factors to different intervention strategies [6–9]. This model is unique in that participants in these studies were required to have no prior history of LBP, and only a percentage of participants developed LBP during the protocol. This enabled the researchers to differentiate between pain developers (PD) and nonpain developers (NPD) and identify factors that differed between the two groups in the early stages of the standing exposure, prior to subjective complaints of LBP [9].

There is evidence that individuals who are predisposed towards LBP development during standing demonstrate altered clinical, biomechanical, and motor control profiles [8–10]. Factors that were consistently found to be important discriminators between PD and NPD individuals were decreased frontal plane control during clinical assessment, elevated cocontraction of the bilateral gluteus medius muscles, decreased resting time (as determined by EMG gaps analysis [11]) for the gluteal muscles, and differences in modulation of trunk flexor/extensor cocontraction during prolonged standing exposure [6, 9]. Preliminary work has also shown that these altered muscle activation profiles can be modified through an active exercise intervention [7]. 

It was unknown how stable these factors might be over time in the absence of any intervention. Therefore, the purpose of the current study was to assess the between-day repeatability of the previously identified factors, as well as commonly utilized clinical assessment measures, within groups of PD and NPD individuals. To accomplish this, a pretest/posttest design was used. This study was conducted as part of a larger intervention study where participants were assigned to participate in an exercise intervention or usual activity (control) groups during an intervening 4-week period. For the current study, only data from the control group was analyzed to determine the stability of the factors that were identified on pretest as being associated with LBP development. There were two primary hypotheses for this study. First, it was expected that individuals would remain in their respective pain development groups during the second standing exposure. Second, it was hypothesized that individuals would demonstrate excellent repeatability of clinical, motor control, and biomechanical factors between the two data collections, as demonstrated by intraclass correlation coefficients (ICCs) of ≥0.80 [12].

## 2. Materials and Methods

Ethics approval for research involving human subjects was obtained from the Office for Research Ethics at the University of Waterloo and written informed consent obtained from all participants prior to their involvement in the study. Twenty-three participants (12 males, 11 females) were recruited from the University of Waterloo and surrounding community populations. Participant characteristics are reported in Table 1. Exclusion criteria included any lifetime event of LBP that was significant enough to seek care from a health care professional or that resulted in greater than 3 days off work or school; current low back or hip pain; previous hip surgery; inability to stand for greater than 4 hours; inability to complete questionnaires; and employment in an occupation requiring extended static standing during the previous 12-months.

### 2.1. Experimental Protocol

Pre- and posttest protocols were identical and have been described in detail previously [9]. Many different measures were assessed on the pretest; however, the focus for this study was on establishing the between-day repeatability of factors that were found to be associated with LBP development on pretesting.

A baseline measure of LBP using a 100-mm visual analogue scale (VAS) with end-point anchors of “no pain” and “worst pain imaginable” was established prior to data collection. A 4-week physical activity scale, the Minnesota Leisure Time Physical Activity Questionnaire (MPAQ) [13] was completed prior to the initial pretest and again before posttest to ensure that there were no significant changes in physical activity during the intervening 4-week period. A licensed physical therapist (ENW) then performed a standardized assessment, identical to what would be done in a clinical setting for a patient presenting with LBP. This assessment included active trunk and hip range-of-motion in all planes (assessed with bubble inclinometer and standard goniometry [14]), lumbar segmental mobility (assessed through posterior-anterior passive mobility testing [14]), active core stability measures (active straight leg raise in supine [15] and active hip abduction in sidelying [16]), assessment of lumbar segmental instability (prone instability test [17]), and trunk muscle endurance tests (time to fatigue in side-bridge [18] and Beiring-Sorenson tests [18]) [9].

Participants were then prepped for surface electromyography (EMG) electrode placement. Disposable pregelled EMG Ag-AgCl electrodes (Blue Sensor, Medicotest, Inc., Olstykke, Denmark) with a 2 cm centre-to-centre inter-electrode distance were applied over 7 bilateral muscle groups: Thoracic Erector Spinae (5 cm lateral to T_9_ spinous process) [19], Lumbar Erector Spinae (above and below L_1_ spinous process) [20], Rectus Abdominis (1 cm above umbilicus and 2 cm lateral to midline) [21], Internal Oblique (1 cm medial to ASIS and beneath a line joining bilateral ASIS) [21], External Oblique (below the ribcage, along a line connecting the inferior costal margin and the contralateral pubic tubercle) [21], Gluteus Medius (2.5 cm distal to the midpoint of the iliac crest) [22], and Gluteus Maximus (midway between the greater trochanter and the sacrum) [22]. All electrode placements were confirmed through palpation and manual resistance. Raw EMG signals were amplified (AMT-8, Bortec, Calgary, Canada; bandwidth = 10–1000 Hz, CMRR = 115 db at 60 Hz, input impedance = 10 GΩ) and collected with a sampling frequency of 2048 Hz using a 16-bit A/D card with a ±2.5 V range. Manual resistance was applied to obtain maximal voluntary contractions (MVCs) for EMG normalization in the following positions: Beiring-Sorensen for trunk extensors [23], prone hip extension for hip extensors, sidelying hip abduction for hip abductors, supine straight-leg curl up, and diagonal curl up to the left and right for trunk flexors [23]. Rest trials were collected in supine and prone positions to determine the resting activation level of the monitored muscles. 

Participants who reported a nonzero VAS score (average 1.85 ± 0.71 mm) following instrumentation had this value subtracted as a bias from the remaining VAS scores collected. Participants were asked to indicate their current level of LBP on the 100 mm VAS every 15 minutes during the 2-hour standing period for a total of 9 VAS scores including the baseline measure. A specific definition or description of LBP was not provided to the participants in an effort to minimize participant expectation.

**Figure 1:**
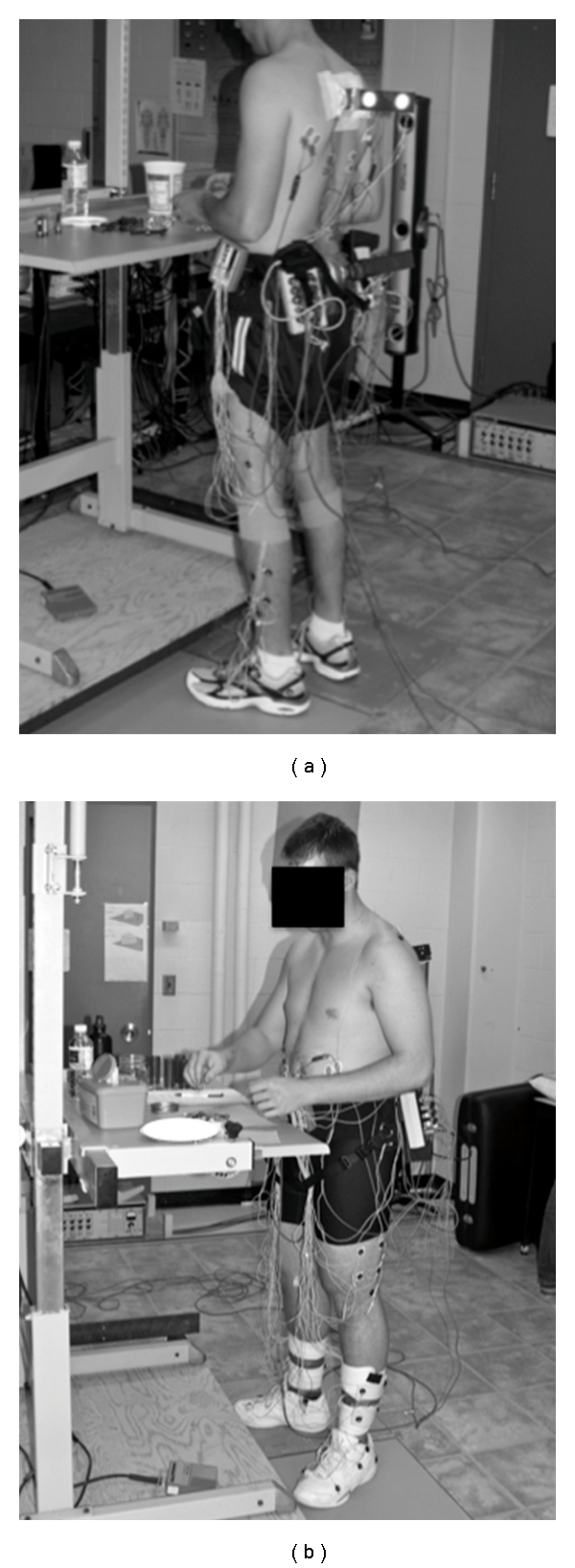
The experimental setup for the prolonged standing protocol.

Participants then entered into the prolonged standing task. The experimental setup is shown in Figure 1. A work surface was positioned in front of the participant and adjusted to a standardized working height with the radial styloid positioned 5-6 cm above the table with the elbow flexed to 90° [24]. Participants were instructed to stand “in their usual manner as if they were standing for an extended period” with the only stipulations being that they could not rest their foot on the standing table frame, and they could not lean on the table surface with their upper extremities to support their body weight. Another baseline VAS was collected just prior to the start of the 2-hour standing period to account for any discomfort that may have developed during the instrumentation period. 

Three different tasks were performed to simulate light occupational activities [9]. These included a “sorting” task, a small object “assembly” task, and a task termed “boredom/waiting” where participants were asked to stand without any activity. Tasks were presented in a block fashion using a random number generator, with 30-minute blocks for each task. EMG data were collected continuously for the 2 hours of standing in 15-minute blocks. 

Participants were classified into PD and NPD groups immediately following the standing protocol based upon their reported LBP scores on the VAS. Based on the Minimal Clinically Important Difference (MCID) of 8 mm for worsening LBP symptoms in a clinical population reported by Hägg et a l. [25], and the relatively low-level pain inducing stimulus used in this study, the decision was made to use a relative increase of 10 mm from baseline on VAS as the cut-point to categorize participants in this study as PD or NPD. 

Signal processing was done through the use of custom programs written in Matlab R2008a, version 7.6.0 (The Mathworks, Inc., Natick, MA, USA). All EMG data underwent a similar algorithm of DC bias removal and bandpass filtering to remove ECG artifact (cutoff frequencies 30–500 Hz) [26] and bandstop (cutoff frequencies 59–61 Hz) [27] for removal of 60 Hz electrical contamination. Following the removal of the noise components, each EMG signal was full-wave rectified and low-pass filtered (dual-pass Butterworth, 4th order, effective cutoff frequency of 2.5 Hz) [28, 29] to create a linear envelope. Resting activity level was subtracted from the EMG signals and signals were normalized to %MVC. EMG data were then downsampled to 32 Hz prior to further analysis as a data reduction measure.

Cocontraction Index (CCI) [30] was used to quantify the level of coactivation between all possible muscle pairs using (1),

(1) $$\text{CCI}=\overset{N}{\underset{i=1}{\sum }}\left(\frac{\text{EM}{\text{G}}_{\text{lo}{\text{w}}_{i}}}{\text{EM}{\text{G}}_{\text{hig}{\text{h}}_{i}}}\right)\left(\text{EM}{\text{G}}_{\text{lo}{\text{w}}_{i}}+\text{EM}{\text{G}}_{\text{hig}{\text{h}}_{i}}\right).$$

The CCI provides a quantitative measure of the degree of coactivation for a pair of muscle groups over a specified number of data points, *N*. “EMG_low_” and “EMG_high_” in the equation are the relative magnitudes of the linear enveloped EMG for the muscle pairs under consideration, *with* “EMG_high_ *”* being the EMG signal with the higher magnitude at each instant in time. As a further data reduction measure, data were collapsed by taking an average of the 15 one-minute window CCI values to yield 8 CCI values for the 2-h standing period for each of the possible 91 muscle pairings. 

An EMG gaps analysis was also performed to determine if there were differences in the amount of rest time for individual muscles during the static standing task. A “gap” was defined as the period of time when the EMG level dropped below 0.5% MVC for a period of 0.2 seconds or longer and is an accepted measure of muscle-resting time [31]. The number of EMG gaps for each monitored muscle, average duration for each gap, and total gap time were calculated for each 15-minute block during the 2-hour standing protocol.

Participants were asked to participate in their usual activities over the 4-week period between data collections. They were requested to refrain from initiating any new exercise programs during this 4-week period.

There was one male participant who was categorized as NPD, who did not complete the posttest for personal reasons. This participant's data was therefore removed from the analysis for the between day comparisons. The final sample distribution is reflected in Table 2. 

### 2.2. Statistical Analyses

Unless otherwise noted, statistical analyses were performed through 3-way general linear models, with between factors of gender, PD/NPD group, and within factor of testing day. To determine the between day repeatability of these measures, intraclass correlation coefficients (ICCs) were computed using a 2-way mixed model for a single examiner. Where significant gender or PD/NPD group differences were detected previously in the general linear models, the ICC was calculated for each gender and/or PD/NPD group separately as appropriate. Bonferroni corrected *P-*values were used for multiple comparisons. Where data were not spherical based on Mauchly's Test, Huynh-Feldt adjusted *P*-values were used to determine significance. Unless otherwise noted, pairwise comparisons were used for *post hoc* testing. Criterion for significance was set *a priori* at *P* < .05. SPSS version 16.0 (SPSS, Inc., Chicago, IL, USA) was used for all statistical analyses.

## 3. Results

### 3.1. Clinical Assessment Findings

There were no significant changes on the majority of the clinical assessment measures between days. According to Shrout and Fleiss [32], ICC values below 0.2 indicate poor, between 0.2 and 0.75 indicate moderate, and above 0.75 indicate excellent agreement. As shown in Table 4, most of the clinical assessment measures had excellent between day repeatability with ICC values ≥0.75 with the exception of hip range-of-motion measurements and examiner-rated Active Hip Abduction (AHAbd) Test scores. Hip extension range of motion was found to have moderate repeatability with ICCs of only around 0.50 for both genders. Examiner-rated AHAbd test scores were found to have moderate to excellent repeatability with ICCs ranging from 0.67 for male PD to 0.92 for male NPD.

### 3.2. Activity Level between Testing Sessions

Minnesota Leisure Time Physical Activity Questionnaire (MPAQ) [13] scores for the 4-week period prior to entering into the study and for the 4-week period in between the two collection days were compared with paired *t*-tests to ensure that activity level for the sample did not change. There were no significant differences detected in activity level (*t_21_* = 1.75, *P* = .10) for the participants, providing confidence that this group was compliant with instructions to continue with their usual level of activity.

### 3.3. Pain Development

**Figure 2:**
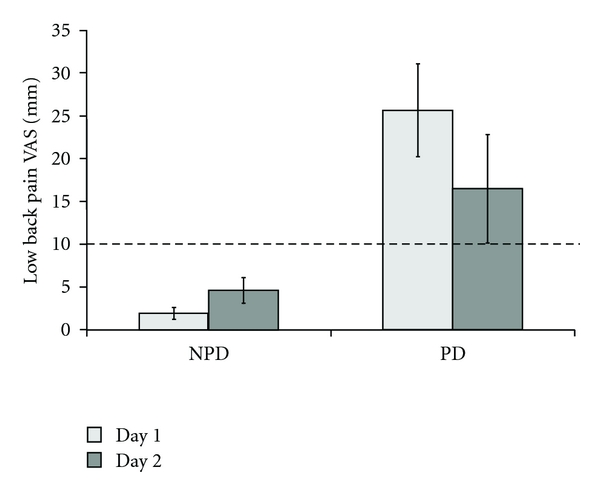
There were no between-day differences for either group in VAS score for the low back during standing. The gray dashed line indicates the VAS cutoff threshold for the PD group classification.

Of the 8 participants who were classified as PD on day 1, 6 (75%) would have been classified as PD on their second testing day, and 2 (25%) would have been classified as NPD based on the criteria of ≥10 mm change from baseline in VAS for the low back. There were 15 participants that were classified as NPD on Day 1. Of these, 2 of the 15 (13.3%) would have been classified as PD on Day 2, and 13 (86.7%) remained in the NPD group on the second testing day. There were no significant differences between Day 1 and Day 2 VAS scores for the PD and NPD groups (*t*_21_ = 1.41, *P* > .05) (Figure 2).

Of the NPD individuals who switched to PD on Day 2, one was female and one was male. Neither participant reported any event that might have caused them to experience pain on the second testing day. The female participant barely exceeded the threshold criteria on posttest with a maximum VAS score of 11 mm. The male participant was well above the threshold criteria with a maximum VAS score of 20 mm.

For the two PD participants who changed to the NPD group on Day 2, one was female and one was male. The female participant reported a VAS score of 0 mm on Day 2 testing and the male participant reported a VAS score of 2 mm on Day 2. The individual between day VAS scores for the participants are shown in Table 3 with the participants who changed groups in bold.

### 3.4. Muscle CoContraction During Prolonged Standing

CCI values for the gluteus medius and trunk flexor/extensor muscles were first entered into 4-way general linear models with between factors of PD/NPD group and gender, and within factors of time (8 repeated measures) and collection day (2 repeated measures). ICC values were then calculated for the 8 repeated measures on each collection day, and if these were found to have low variability (i.e., large ICC values), these 8 repeated measures were then averaged to yield a single CCI average value for each day. Between day ICC values were then calculated using the average CCI values for each collection day. The between day ICC values were calculated for the entire sample, and also for the PD and NPD groups separately.

There were no significant between-day differences (*P* > .05) detected for gluteus medius CCI in the general linear model. As shown in Tables 5 and 6, within-day repeatability was excellent for both collection days, and between-day repeatability was also excellent for gluteus medius CCI. 

There was a main effect of collection day (*F_1,17_* = 4.831, *P* < .05) for trunk flexor/extensor CCI, with individuals having an overall decrease in CCI between collection days, 2184 ± 229% MVC on Day 1 to 1626 ± 196% MVC on Day 2. As with the gluteus medius CCI, within and between day repeatability was excellent for the combined PD/NPD groups (Table 5). As can be seen in Table 6, between-day repeatability was lower in the NPD group. When the PD/NPD groups were separated, the PD group was found to be very consistent in their between-day trunk cocontraction patterns while the NPD group was found to be very dissimilar between the collection days.

### 3.5. Total Gap Length During Prolonged Standing

The primary measure on the EMG gaps analyses that was found to be predictive of LBP during standing previously was the total gap length for each 15-minute window over the 2-hour standing exposure, for the following six muscles: right external oblique (REO), left internal oblique (LIO), right gluteus medius (RGMed), left gluteus medius (LGMed), right gluteus maximus (RGMax), and left gluteus maximus (LGMax). These values were entered into 4-way general linear models as previously described, and ICC values calculated to determine within-day and between-day repeatability. 

There were no significant between day effects in total gap length detected for the REO, LIO, RGMed, LGMed, or LGMax muscles. There was a significant gender by collection day interaction (*F_1,17_* = 5.21, *P* < .05) in total gap length for the RGMax muscle. Males had an average increase in total gap length (from 563.0 ± 74 to 647.8 ± 85 seconds) and females had an average decrease (from 563.0 ± 69 to 470 ± 79 seconds) between the collection days. As can be seen in Table 7, the within-day and between-day repeatability was moderate to excellent for the total gap length for all of the muscle groups under consideration.

## 4. Discussion

The between-day repeatability of the assessed outcome measures was, in general, excellent. While not all of the participants remained in their initial PD/NPD groups on the second day of testing, the majority (83%) of them did, supporting the first hypothesis. It seems that individuals who are predisposed to develop LBP during a standing exposure remain fairly consistent in this response. The cutoff threshold of ≥10 mm change in VAS to be considered PD was chosen *a priori* to this data collection. It was expected that participants would remain within their original PD/NPD group with repeated testing in the absence of an intervention being applied. This was true for the majority of the participants. Neither of the two NPD participants who changed over to the PD groups on Day 2 reported zero VAS scores on Day 1. It is possible that if the standing exposure had been longer than 2hours, these two individuals might have been classified as PD on the first collection day. The two PD participants who changed over to the NPD group on Day 2 both had VAS scores that were just over the threshold criteria for classification into the PD group on Day 1. Although there were other participants with scores in those ranges that did not change groups on Day 2, it may be that individuals who are close to that threshold criteria, which was set somewhat arbitrarily based on reports in the literature for other pain conditions, are more fluid in their day-to-day patterns. The rest of the participants remained in their Day 1 groups and appear to be more consistent in their predisposition for experiencing, or not experiencing, pain when exposed to prolonged standing.

The between-day repeatability of the assessment measures was generally excellent, with the exception of hip extension range of motion and the examiner-rated hip abduction test. Between-day measures of hip extension were poor for both genders. Hip flexion measurements had fair between-day repeatability for males. For the between day differences in males for hip flexion, it is probable that there were actual changes within individuals given that standard goniometric techniques were used for all range of motion measures and there were no differences detected in the females. It is unlikely that the examiner would have introduced a systematic error in this measurement in a single group. Intrarater ICC values for hip goniometric measurements have been reported in the literature previously. Holm and colleagues [33] found intrarater ICC values to range from 0.80 to 0.94 for hip flexion, extension, and internal and external rotation. Other researchers have reported lower intrarater reliability scores for hip extension (ICC = 0.56) and external rotation (0.58) [34]. Given this wide range of reported intrarater ICC values for hip extension, it is likely that the between day differences observed in this study are a function of examiner error rather than variability in the sample.

Participants were highly repeatable in their self-assessment of AHAbd Test [16] difficulty, while the examiner-rated score had moderate to excellent repeatability for PD groups. Whether this was due to actual differences in the individuals' test performance or was a reflection of poor intrarater reliability is difficult to say. There was a single examiner (ENW) for this study, and the examiner was no longer blinded to the participant's PD/NPD group on the second day. For measures incorporating potential subjective examiner bias, this presents a limitation for these measures. However, the fact that the male PD group had higher average scores on Day 2 (indicating poorer test performance) and the female PD group had lower Day 2 scores (indicating better test performance) tends to refute this. As this is a new test in the very initial stages of development, systematic inter- and intrarater reliability studies need to be conducted on it beyond this single examiner small sample-size initial study. As noted previously, it is unclear whether this is a reflection of true day-to-day variability in the participants, or due to variability within the examiner. Because the clinical assessment includes interaction with an examining individual, and in the case of the AHAbd test requires a judgment to be made by the examining individual, this variability cannot be separated. 

Muscle activation patterns during prolonged standing were very repeatable, with very few between-day differences noted. For cocontraction of the gluteus medius muscles, ICC values exceeded 0.80. For cocontraction of the trunk flexor/extensor muscles, between-day ICC for the PD group was very good (ICC > 0.80); however, it was poor for the NPD group (ICC < 0.10). This indicates that there is more day-to-day variability in trunk muscle coactivation in individuals who are not predisposed to develop LBP during standing, while pain developers tend to utilize the same muscle coactivation pattern more consistently. This is consistent with reports in the literature that people with LBP have decreased variability in muscle onsets of the internal oblique with a self-initiated arm-raise perturbation [35]. There have been similar findings in healthy individuals who have had acute, experimentally induced LBP (hypotonic saline injection) [36]. The conclusions that have been made from these studies are that people with LBP have a limited number of strategies they can draw upon, thereby limiting their ability to adapt to changing physical demands and circumstances. The other measures of muscle activation patterns during standing that were previously found to have PD/NPD group differences (average EMG levels and total Gap length) [9], were all very repeatable between days with ICC values ranging from 0.62 to 0.87 for the control groups. The hypothesis that there would be good between-day repeatability for factors associated with LBP development during standing was largely supported, as most of the variables were found to have good-to-excellent between-day ICC values. These findings greatly increase confidence that any observed changes in these measures in response to intervention were truly related to the intervention rather than due to natural between-day variability.

## 5. Conclusions

The purpose of this paper was to determine the normal day-to-day variability in clinical findings, motor control, and muscle activation profiles in people who have been classified as PD and NPD during an exposure that has been designed to functionally induce LBP. Factors that have been previously determined to be of importance in discriminating between PD/NPD individuals were found to be highly stable between days when physical activity was maintained at a consistent level, with the exception of trunk flexor/extensor cocontraction. These findings are important in that they increase confidence in attributing clinical test results, motor control, and muscle activation profile changes following intervention as being directly related to the intervention. The repeatability of the PD/NPD classification based on VAS score is also important in that it provides further support for the utility of this functionally induced LBP protocol as a model for studying LBP development using a short-duration, prospective methodology.

## Figures and Tables

**Table 1:** Baseline characteristics of participants.

Group Statistics						
	Group	*N*	Mean	Std. Deviation	Std. Error Mean	Independent *t*-test *P*-value
Age (years)	NPD	**15**	22.6	3.3	0.85	.75
	PD	**8**	22.1	2.9	1.0	

BMI (Kg/m^2^)	NPD	**15**	23.70	2.90	0.75	.40
	PD	**8**	24.92	3.78	1.34	

Minnesota Leisure Time Physical Activity Score	NPD	**15**	15406.6	7596.9	1961.5	.15
	PD	**8**	14060.4	5188.4	1834.4	

Baseline VAS (mm)—Low Back	NPD	**15**	0.93	1.94	0.50	.14
	PD	**8**	0.63	0.74	0.26	

**Table 2:** Sample distribution among groups following participant dropout.

		*n*	Total* n *
Pain Developers (PD)	Male	3	**8**
	Female	5	

NonPain Developers (NPD)	Male	8	**14**
	Female	6	

Total			**22**

**Table 3:** Low back Visual Analog Scale (VAS) scores for Days 1 and 2. Participants who changed groups are in bold.

Participant ID	Day 1 VAS	Group Day	Day 2 VAS	Group Day	Change
	(mm)	1	(mm)	2	(mm)
**F02**	**5 **	**NPD**	**11 **	**PD**	**+6 **
F05	0	NPD	0	NPD	0
F09	0	NPD	0	NPD	0
F10	0	NPD	0	NPD	0
F14	6.5	NPD	0	NPD	−6.5
F18	6	NPD	3	NPD	−3
M02	0	NPD	6.5	NPD	+6.5
M05	4.4	NPD	0	NPD	−4.5
M06	0	NPD	0	NPD	0
M11	0	NPD	0	NPD	0
M14	0	NPD	0	NPD	0
**M16**	**6 **	**NPD**	**20 **	**PD**	**+14 **
M18	0	NPD	0	NPD	0
M19	1.5	NPD	0	NPD	−1.5
F11	25	PD	24	PD	−1
F12	32	PD	10	PD	−22
**F16**	**14 **	**PD**	**0**	**NPD**	−**14 **
F19	16	PD	12	PD	−4
F21	13	PD	20	PD	+7
**M08**	**12.5 **	**PD**	**2 **	**NPD**	−**10.5 **
M09	37	PD	10	PD	−27
M22	56	PD	56	PD	0

**Table 4:** Between-day repeatability for clinical assessment tools.

Assessment tool			ICC value
Lumbar Flexion Range of Motion (ROM)			0.94
Lumbar Extension (ROM)	Male		0.93
	Female		0.92

Lumbar Lateral Flexion (ROM)			0.80
Hip Flexion (ROM)	Male		0.61
	Female		0.70
Hip Extension (ROM)	Male		0.48
	Female		0.50

Hip Internal Rotation (ROM)			0.91
Hip External Rotation (ROM)			0.74
Straight Leg Raise (ROM)			0.87
Active Straight Leg Raise (ASLR) Test			0.79

Self Rated AHAbd Test	NPD		0.87
	PD		0.85

Examiner Rated AHAbd Test	Male	NPD	0.92
		PD	0.67
	Female	NPD	0.83
		PD	0.77

Extensor Endurance Time	Male		0.88
	Female		0.69

Side Support Time			0.91
4-week Activity Level (MPAQ)			0.90

**Table 5:** Within-day repeatability for gluteus medius and trunk flexor/extensor cocontraction index during standing.

	Day 1	Day 2
	ICC	ICC
Gluteus Medius CCI	0.95	0.92
Trunk Flexor/Extensor CCI	0.89	0.94

**Table 6:** Between-day repeatability for trunk flexor/extensor and gluteus medius cocontraction index during standing. Poor repeatability is indicated in *bold italic*.

	Gluteus Medius CCI	Trunk Flexor/Extensor CCI
	Between Days ICC	Between Days ICC
PD/NPD Combined	0.89	0.52
NPD	0.87	***0.09 ***
PD	0.90	0.82

**Table 7:** Within-day and between-day repeatability for total gap length during standing.

Muscle Group	Day 1	Day 2	Between Days
	ICC	ICC	ICC
R External Oblique	0.98	0.98	0.67
R Gluteus Medius	0.97	0.98	0.62
R Gluteus Maximus	0.94	0.97	0.87
L Internal Oblique	0.98	0.98	0.73
L Gluteus Medius	0.96	0.95	0.66
L Gluteus Maximus	0.96	0.98	0.86
